# KCQI: Novel Index for Assessment of Comprehensive Quality of Kiwifruit During Shelf Life Using Hyperspectral Imaging and One-Dimensional Convolutional Neural Networks

**DOI:** 10.3390/foods14223886

**Published:** 2025-11-13

**Authors:** Yongxian Wang, Kaisen Zhang, Yi Liu, Junsheng Liu, Ruofei Liu, Bo Ma, Linlin Sun, Linlong Jing, Xinpeng Cao, Hongjian Zhang, Jinxing Wang

**Affiliations:** 1 College of Mechanical and Electrical Engineering, Shandong Agricultural University, Taian 271018, China; wyongxian@163.com (Y.W.); zhangkaisen0313@163.com (K.Z.); 2021010101@sdau.edu.cn (Y.L.); 15505487223@163.com (J.L.); 19860912870@163.com (R.L.); mabo_ok@163.com (B.M.); sunlinlin@sdau.edu.cn (L.S.); jinglinlong@sdau.edu.cn (L.J.); caoxinpeng@sdau.edu.cn (X.C.); 2 Shandong Key Laboratory of Intelligent Production Technology and Equipment for Facility Horticulture, Taian 271018, China; 3 Shandong Engineering Research Center of Agricultural Equipment Intelligentization, Taian 271018, China

**Keywords:** *Actinidia* spp., postharvest life, comprehensive quality, 1D-CNN, prediction model

## Abstract

Non-destructive assessment of kiwifruit quality is critical for postharvest preservation and grading. This paper proposes a novel quantitative evaluation method for the kiwifruit comprehensive quality index (KCQI) during shelf life, based on hyperspectral imaging (HSI) combined with a one-dimensional convolutional neural network (1D-CNN). Hyperspectral images of two kiwifruit cultivars were acquired at four shelf-life stages using an HSI system, and six quality parameters were measured as reference standards. Based on correlation and factor analyses, five key parameters—soluble solids content, firmness, L*, b*, and chroma—were selected to construct the KCQI. Three spectral band selection methods and three modeling algorithms were compared, with the competitive adaptive reweighted sampling (CARS)–1D-CNN model yielding the highest prediction accuracy ($R_{P}^{2}$ = 0.82, RMSEP = 0.26, RPD_P_ = 2.39). Subsequently, a spatial distribution map was generated to visualize the KCQI. These results demonstrate the potential of the HSI–1D-CNN approach for accurate postharvest quality monitoring and intelligent grading.

## 1. Introduction

Kiwifruit (*Actinidia* spp.) is renowned for its high vitamin C content, dietary fiber, and various antioxidants, and has become one of the fastest-growing fruit categories worldwide [1]. As a typical climacteric fruit, kiwifruit softens through cell wall degradation and increases in sweetness via starch hydrolysis to soluble sugars during postharvest storage and shelf life [2,3]. Accurate, non-destructive monitoring of these quality changes is essential for maintaining market value and optimizing supply chain performance. Traditional assessments rely on subjective distributor evaluations or laboratory physicochemical analyses; the former suffers from poor repeatability and high subjectivity, while the latter is destructive, time-consuming, and unsuitable for real-time, non-destructive monitoring [4,5]. Therefore, the development of rapid, non-destructive methods for the real-time quality evaluation of kiwifruit during shelf life is crucial for improving grading accuracy and extending postharvest longevity.

In recent years, hyperspectral imaging (HSI) has emerged as a research hotspot for non-destructive postharvest quality assessment of fruits and vegetables, owing to its “spectral-image integration” advantage [6,7]. Several studies have reported stage-wise progress in monitoring kiwifruit quality during shelf life. For example, Lee et al. acquired hyperspectral images of kiwifruit at five storage periods, establishing prediction models for soluble solid content (SSC) and firmness through second-derivative preprocessing coupled with support vector regression, achieving prediction set coefficients of determination ($R_{P}^{2}$) values of 0.940 and 0.878, respectively [8]. Hu et al. determined the HSI of seven storage-age kiwifruit fruits treated with 1-methylcyclopropene, and constructed prediction models for glucose, fructose, and sucrose using multiple linear regression algorithm, with prediction set coefficients of determination ($R_{P}^{2}$) of 0.934, 0.867, and 0.705, respectively [9]. Furthermore, Shang et al. employed the HSI combined with chemometric methods to estimate kiwifruit quality: they used competitive adaptive reweighted sampling (CARS) to select spectral bands correlated with SSC and firmness, and then built multiple linear regression models yielding validation $R_{P}^{2}$ values of 0.896 and 0.871 and relative predictive deviations (RPDs) of 3.12 and 2.81 for SSC and firmness, respectively [10]. Although the above studies verified the predictive ability of the HSI on kiwifruit quality, they have predominantly focused on single parameters, failing to reveal synergistic changes among multiple quality parameters during shelf life and lacking exploration of integrating these parameters into a comprehensive evaluation index.

Meanwhile, with the rapid development of artificial intelligence, deep learning techniques—exemplified by convolutional neural networks (CNNs)—have demonstrated outstanding performance in quantitative analysis of spectral data, offering new avenues for processing HSI datasets. For instance, Zheng et al. combined the HSI with a one-dimensional CNN (1D-CNN) to build a SSC prediction model for apples, achieving a prediction-set coefficient of determination ($R_{P}^{2}$) of 0.90 and an RMSEP of 0.37%, outperforming a PLSR-based model ($R_{P}^{2}$ = 0.88, RMSEP = 0.44%) [11]. Wang et al. acquired hyperspectral images of apples using an HSI sensor, extracted SSC-related spectral bands via the successive projections algorithm (SPA), and developed a 1D-CNN–based SSC prediction model; this approach attained the best performance among chemometric methods, with calibration and prediction R^2^ values of 0.845 and 0.808, respectively [12]. Xu et al. integrated the HSI with CNN to predict SSC in pear, achieving higher accuracy than traditional PLSR and SVR models, further validating the reliability of CNNs for high-precision quality prediction [13]. Although these studies highlight the potential of HSI–CNN fusion for fruit quality estimation, a quantitative evaluation method specifically targeting the comprehensive quality of kiwifruit during shelf life remains undeveloped.

**Figure 1:**
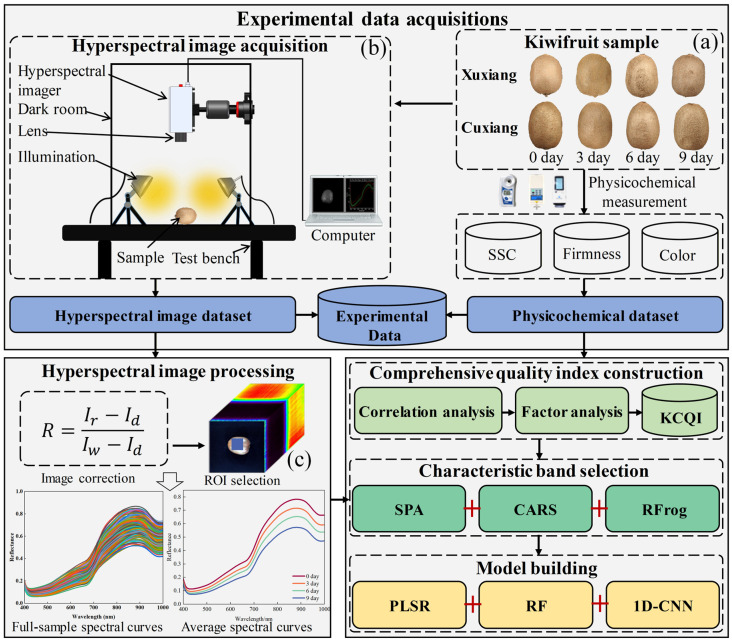
Flowchart of this study: (**a**) Kiwifruit samples; (**b**) Hyperspectral imaging system; and (**c**) Selection of the region of interest.

To address this gap, this paper aims to propose a novel kiwifruit comprehensive quality index (KCQI) during shelf life that integrates multiple quality parameters and develops a quantitative estimation method for the KCQI by combining the HSI with 1D-CNN. The specific objectives are as follows: (1) to construct a novel KCQI based on the synergistic integration of multiple quality parameters, (2) to develop a quantitative prediction model for the KCQI using selected spectral characteristic bands in conjunction with 1D-CNN, and (3) to realize the pixel-level mapping and spatial distribution visualization of the KCQI to reveal the law of quality evolution. Figure 1 shows the overall flowchart of this study.

## 2. Materials and Methods

### 2.1. Samples

Two kiwifruit cultivars (‘Xuxiang’ and ‘Cuixiang’) (Figure 1a) were purchased on 15 November 2024 from the fruit wholesale market in Taian, Shandong Province, China. The fruits of both cultivars were obtained from the same delivery batch at the market. Immediately after purchase, all fruits were transported on the same day to the Postharvest Processing Laboratory of Shandong Agricultural University. Upon arrival, the fruits were held at ambient temperature for 3 h, after which samples exhibiting uniform shape, normal coloration, and no visible mechanical injury were selected. The selected fruits were sorted by cultivar and then arranged in ventilated polyethylene boxes (six fruits per box) and stored at 18 ± 2 °C to simulate typical retail shelf conditions. A total of 120 fruits per cultivar (240 total) were selected. At four storage times—day 0 (purchase day), day 3, day 6, and day 9—thirty fruits of each cultivar were randomly sampled for hyperspectral image acquisition and physicochemical analyses.

### 2.2. Hyperspectral Imaging System

Hyperspectral images of the kiwifruit samples were acquired using a GaiaField-V10E system (Dualix Spectral Imaging Technology Co., Ltd., Wuxi, Jiangsu, China). The system comprised a hyperspectral camera (GaiaField-V10E), two 200 W tungsten–halogen lamps (HSIA-LS-T-200W), an objective lens (HSIA-OL23), a standard white reference panel (HSIA-CT-150 × 150), a dark chamber, and a computer equipped with SpecView(version 2.9.3.3) data acquisition software. The system operated over a spectral range of 400–1000 nm with a spectral resolution of 2.8 nm and employs a detector array of 1392 × 1040 effective pixels to generate hyperspectral cubes consisting of 256 spectral bands.

### 2.3. Hyperspectral Image Acquisition and Correction

Kiwifruit samples were removed from the storage boxes and allowed to equilibrate to ambient temperature until their surfaces were free of condensation. Each fruit was then placed on the stage of a light-proof imaging chamber, ensuring that the pedicel was parallel to the stage surface (Figure 1b). To optimize image clarity and avoid overexposure, the camera exposure time and gain were set to 3.67 ms and 5 dB, respectively, with a fixed vertical distance of 46 cm between the lens and the sample. Prior to sample imaging, a standard white reference image ($I_{w}$) and a dark background image ($I_{d}$) (acquired with the lens capped) were acquired for radiometric correction using Equation (1):

(1) $$R_{c}=(I_{r}-I_{d})/(I_{w}-I_{d})$$

where $I_{r}$ and $R_{c}$ denote the raw hyperspectral image and corrected reflectance image.

Corrected reflectance images were imported into ENVI 4.6 (Research Systems Inc., Boulder, CO, USA), where a 110 × 110-pixel region of interest (ROI) was manually delineated on each hyperspectral cube to encompass the principal scanned area of the fruit (Figure 1c). The average reflectance value of all pixels within the ROI was calculated and extracted as the representative spectral data for each fruit, serving as input for subsequent KCQI quantitative model development.

### 2.4. Physicochemical Measurement

#### 2.4.1. Color

After HSI, a 1 cm^2^ section of peel was removed from the equatorial region of each kiwifruit using a fruit knife. The color parameters (L*, a*, b*) of the exposed flesh were measured with a color reader (CR-10Plus, Konica Minolta, Inc., Tokyo, Japan), and the chroma value was calculated [14]. Measurements were taken at four measurement points around the fruit, spaced 90° apart, and the average of these four readings was reported as the reference color value.

#### 2.4.2. Firmness

Following color measurements, flesh firmness was determined using a digital fruit firmness tester (GY-4, Sanliang Technology Co., Ltd., Guangzhou, China) equipped with a 3.2 mm diameter stainless steel probe. The probe was vertically penetrated into the flesh at an acceleration rate of 5 mm/s until a penetration depth of 10 mm was achieved. The maximum force value displayed by the instrument at this depth was recorded. The average firmness value from four measurement points was adopted as the reference firmness value.

#### 2.4.3. Soluble Solids Content

After firmness testing, flesh samples were collected from the four measurement sites and juice was extracted using a juice extractor. A few drops of the freshly expressed juice were placed on the prism plate of a digital refractometer (PAL-1, ATAGO, Tokyo, Japan), and the SSC was measured. The average of four measurements was reported as the reference SSC value.

### 2.5. Correlation and Factor Analysis

To construct the novel comprehensive quality index, correlation and factor analyses were performed on the kiwifruit quality dataset. First, Pearson correlation analysis was conducted to investigate the interrelationships among individual quality parameters. Next, parameters exhibiting significant correlations were normalized—each variable was mapped to a common scale to eliminate the effects of differing units. Finally, factor analysis was applied to extract the underlying principal factors from the full set of parameters, thereby laying the groundwork for constructing the new composite index.

In the factor analysis, data suitability was first evaluated by the Kaiser–Meyer–Olkin (KMO) measure and Bartlett’s test of sphericity. A KMO value greater than 0.5 indicated that the dataset was adequate for factor analysis, and a Bartlett’s test significance level of *p* = 0.00 confirmed that correlations among variables were sufficiently strong to proceed with factor extraction [15,16]. The number of factors retained was determined by cumulative variance contribution: factors accounting for a cumulative contribution of ≥80% were selected to define the optimal feature space [17]. Finally, representative quality parameters were chosen based on their loadings in the rotated component matrix. Parameters with absolute loadings greater than 0.5 and no significant cross-loadings were considered optimal. These selected parameters were then linearly combined, weighted by their respective factor loadings, to construct a novel comprehensive quality index. All correlation and factor analyses were performed in IBM SPSS Statistics 26 (SPSS Inc., Chicago, IL, USA).

### 2.6. Quantitative Prediction Model Development and Evaluation

#### 2.6.1. Sample Set Division and Characteristic Band Selection

The full dataset was divided into a calibration set and a prediction set at an 8:2 ratio using the sample set partitioning based on joint X–Y distances (SPXY) algorithm. The SPXY approach considers both the distribution of the spectral predictors (X) and the target KCQI values (Y) during splitting, ensuring that the calibration and prediction sets are consistent in both spectral and response-value spaces, thereby enhancing model generalizability and prediction stability [18].

To mitigate multicollinearity and improve computational efficiency, three band-selection algorithms were applied to the hyperspectral bands: the SPA, CARS, and the random frog (RFrog) method. SPA, a forward-projection technique, iteratively removes redundant bands by minimizing projection residuals in the residual subspace, yielding a minimally redundant yet highly discriminative band subset that alleviates multicollinearity and stabilizes the model [19]. CARS constructs sampling weights based on the absolute values of PLS regression coefficients and employs an iterative weighted sampling strategy to dynamically adjust each band’s selection probability, preferentially retaining bands that contribute most to predictive performance [20]. RFrog, inspired by reversible-jump Markov chain Monte Carlo, evaluates the selection probability of each band in conjunction with the PLS model’s minimum cross-validation root-mean-square error (RMSECV) criterion to determine the optimal number of bands [21]. All the aforementioned characteristic selection methods were executed strictly on the calibration set defined by SPXY to ensure the integrity of model evaluation and prevent data leakage.

#### 2.6.2. Model Development and Evaluation

**Figure 2:**
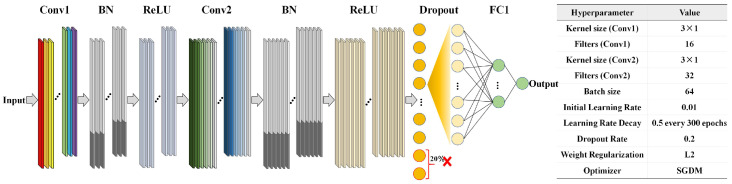
1D-CNN model network structure demonstration.

Using the raw reflectance values of kiwifruit as input, quantitative prediction models for the KCQI were developed using PLSR, random forest (RF), and 1D-CNN. PLSR identifies an orthogonal set of latent variables that maximizes the explained variance in the spectral data while optimizing covariance with the KCQI [22]. During modeling, 10-fold cross-validation was used to select the optimal number of latent variables and prevent overfitting. RF, a nonparametric ensemble method, constructs numerous decision trees on random subsets of samples and features, averaging their outputs to improve prediction stability and noise robustness; the number of trees was set to 100 and the minimum leaf-node size to 8 to balance bias and variance [23]. The 1D-CNN model automatically extracts multilevel local spectral features via one-dimensional convolution kernels [24]. As illustrated in Figure 2, the network comprises two convolutional layers (Conv1 and Conv2), each followed by batch normalization (BN) and ReLU activation layers, to accelerate convergence and introduce nonlinearity. A Dropout layer with a 20% drop rate was inserted after the second ReLU to suppress overfitting. A fully connected layer (FC1) then aggregates the multichannel feature maps into a single output, and a regression layer computes the semi-mean-square error to yield continuous KCQI predictions.

Model performance was assessed by the calibration set coefficient of determination ($R_{C}^{2}$), prediction set coefficient of determination ($R_{P}^{2}$), RMSEC, RMSEP and the RPD_P_ [12,25]. All modeling and evaluation procedures were carried out in MATLAB 2021 (MathWorks Inc., Natick, MA, USA).

## 3. Results and Discussion

### 3.1. Spectral Characteristics of Kiwifruit

**Figure 3:**
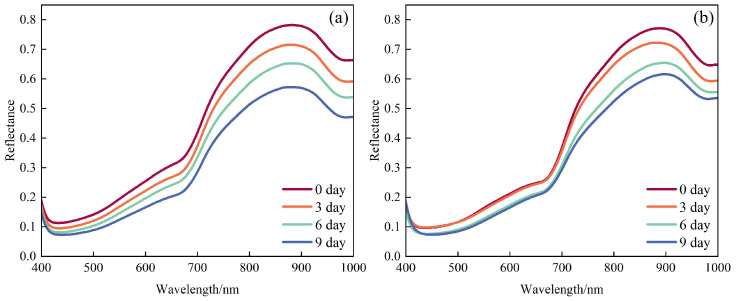
Average reflectance spectra of kiwifruit at different shelf life stages: (**a**) ‘Xuxiang’ and (**b**) ‘Cuixiang’.

Figure 3 shows the average reflectance spectra (400–1000 nm) of ‘Xuxiang’ and ‘Cuixiang’ kiwifruit at shelf life stages of 0, 3, 6, and 9 days. An absorption feature around 420 nm is attributed to chlorophyll a and b absorption in the blue region [12]. The absorption peak at 670 nm corresponds to the characteristic red-light absorption of chlorophyll a; its amplitude decreased with storage time, indicating chlorophyll a degradation. In the near-infrared (NIR) region, reflectance at 830 nm declined continuously over the shelf life, reflecting both reduced scattering due to softening of cell walls and enlargement of intercellular spaces and enhanced NIR absorption resulting from accumulation of soluble solids [26]. The pronounced absorption near 970 nm arises from the strong second overtone of the O–H vibration in water molecules, and changes in its depth directly indicate dynamic regulation of fruit moisture content during storage [27].

### 3.2. Statistics and Analysis of Reference Values

**Figure 4:**
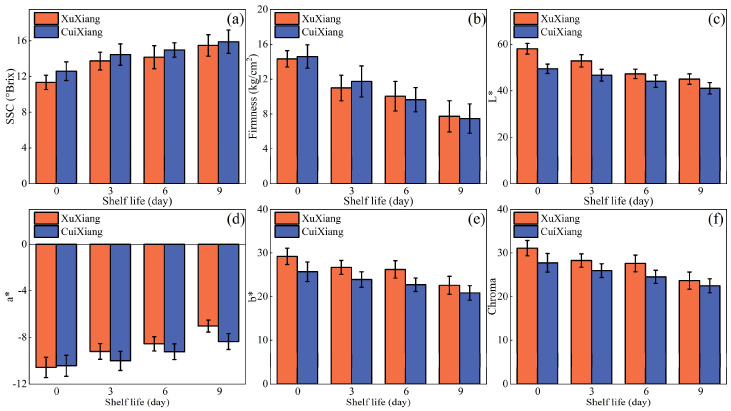
Statistical analysis of changes in kiwifruit quality indicators over increasing shelf life. (**a**) SSC, (**b**) Firmness, (**c**) L*, (**d**) a*, (**e**) b* and (**f**) Chroma. Error bars represent the standard deviation (SD).

Figure 4 shows changes in kiwifruit quality parameters during shelf life. As storage time increased, the SSC of both cultivars increased from approximately 12 °Brix to 16–17 °Brix, and the a* value increased from about −9 to −6, both exhibiting a continuous upward trend. In contrast, L* values decreased from around 55 to 45, b* values from about 30 to 22, and chroma from approximately 32 to 24, all showing continuous declines. Meanwhile, fruit firmness fell from an initial 13–14 kg cm^−2^ to below 8 kg cm^−2^, reflecting moisture loss and textural changes resulting from cell-wall softening and tissue relaxation during storage. These trends indicate that, as storage time increased, soluble solids accumulate and pigmentation shifts toward red, while lightness, color saturation, and firmness decrease in parallel—providing the data basis for constructing a comprehensive quality index.

### 3.3. Correlation Analysis Results

**Figure 5:**
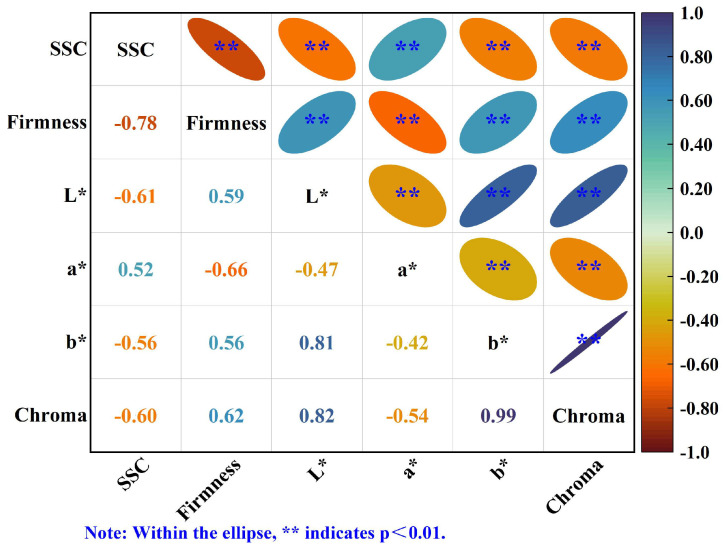
Pearson correlation matrix of kiwifruit quality parameters during shelf life.

Pearson correlation coefficients among the quality parameters are displayed in Figure 5. SSC exhibited a significant positive correlation with a* (r = 0.52) and significant negative correlations with firmness, L*, b*, and chroma (r = −0.78, −0.61, −0.56, and −0.60, respectively). Firmness was positively correlated with L*, b*, and chroma (r = 0.59, 0.56, and 0.62, respectively) but negatively correlated with a* (r = −0.66). Additionally, L* correlated positively with b* and chroma, while a* correlated negatively with b* and chroma; b* and chroma exhibited a very strong positive correlation (r = 0.99). These interrelationships indicate that, as storage time increased, increases in soluble solids are concomitant with reddening of the peel (higher a*) and tissue softening (lower firmness), whereas lightness and color saturation metrics decline in tandem. These correlation patterns not only elucidate the intrinsic linkages underlying kiwifruit quality changes during storage but also inform the selection of representative parameters for subsequent factor-analysis–based index development.

### 3.4. Factor Analysis Results and Construction of the KCQI

**Figure 6:**
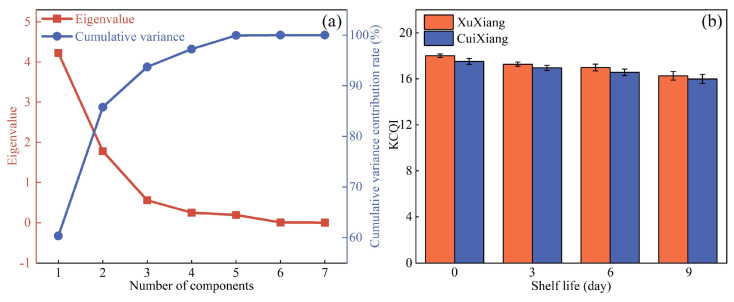
(**a**) Scree plot and cumulative variance contribution rates after factor analysis; (**b**) KCQI trend over shelf life. Error bars represent the SD.

After normalizing all kiwifruit quality parameters, the dataset’s suitability for factor analysis was first evaluated. The KMO measure was 0.66, indicating that sampling adequacy met the requirements for factor analysis. Bartlett’s test of sphericity yielded a significance level of *p* = 0.000, demonstrating that the correlation matrix significantly deviated from an identity matrix and was therefore appropriate for extracting common factors. These results confirmed that the data were suitable for exploratory factor analysis. As shown in Figure 6a, the scree plot and cumulative variance contribution rates indicated that the first three factors accounted for more than 90% of the total variance. Therefore, these three factors were considered to capture the majority of the information contained in all quality parameters, exhibiting strong internal consistency and explanatory power.

To identify the original variables most strongly associated with these factors, we examined the factor-loading matrix (Table 1). Factor 1 was primarily loaded by soluble SSC, firmness, L*, and chroma; Factor 2 was dominated by b*; no variable exhibited sufficient loading on Factor 3, so it was excluded from the final evaluation framework. Since Factors 1 and 2 together explained 84.9% of the total variance, they were deemed to represent the major variation in the original quality parameters. Consequently, five key parameters were selected as comprehensive evaluation factors.

Based on the principle of using positive loadings in the numerator and negative loadings in the denominator, the KCQI was defined as follows:

(2) $$\mathrm{KCQI}=\ln[\frac{1000\times F\times L*\times b*\times C}{\mathrm{SSC}}]$$

where *F* denotes firmness and C denotes chroma.

Applying this formula to weight and integrate the selected quality parameters yielded the KCQI trend over the shelf life period (Figure 6b). At the early shelf-life stage, higher KCQI values indicated firmer, vibrant-colored fruit with lower SSC, corresponding to a crisp texture and higher acidity. As shelf life progressed, the gradual decrease in the KCQI reflected fruit softening, darkening color, and increased SSC, which associated with a soft texture and elevated sweetness. This composite index integrates both structural and compositional attributes of kiwifruit, providing a quantitative foundation and theoretical support for comprehensive quality assessment.

### 3.5. Characteristic Band Selection

**Figure 7:**
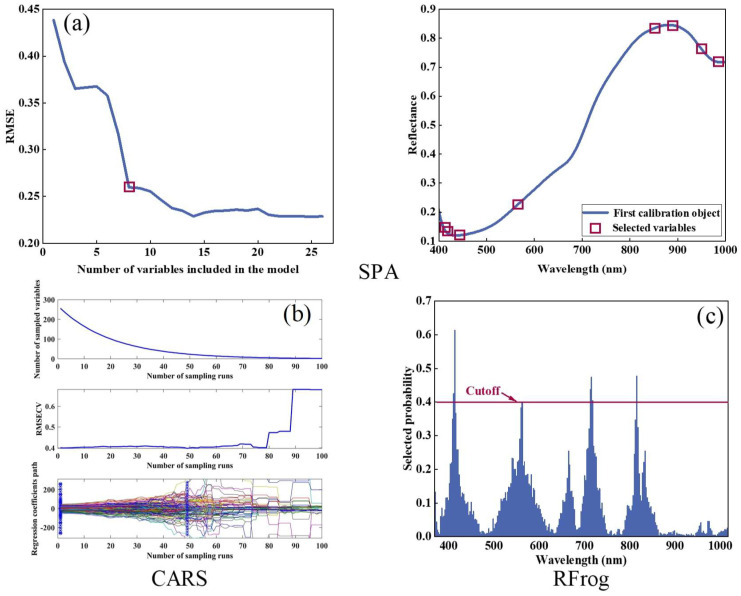
Characteristic band selection. (**a**) SPA selection process, showing RMSE decreasing as the number of bands increases; (**b**) CARS selection process, with the number of retained bands decreasing as the number of sampling iterations increases and RMSECV varying dynamically; (**c**) RFrog selection probabilities for each band, with bands above the threshold line being selected.

Characteristic band selection was performed using SPA, CARS and RFrog (Figure 7). In Figure 7a, the SPA procedure is illustrated: as the number of bands increased, the RMSE decreased continuously; when RMSE reached 0.2596, an F-test at significance level α = 0.25 indicated no significant difference from the minimum RMSE, resulting in eight characteristic bands. Figure 7b shows the CARS process: as Monte Carlo sampling iterations increased, the number of retained bands decreased; at 50 iterations, RMSECV attained its minimum value of 0.3972, corresponding to 19 characteristic bands. Figure 7c presents the selection probabilities for each band calculated by RFrog: when RMSECV reached its lowest value of 0.3373, a probability threshold of 0.40 was applied, yielding seven characteristic bands. The exact bands selected by each algorithm are listed in Table 2.

### 3.6. Model Development for KCQI Prediction

Using the selected characteristic bands, three predictive models for the KCQI were constructed based on PLSR, RF, and 1D-CNN algorithms. Table 3 summarizes each model’s performance metrics on both the calibration and prediction sets. Among them, the model combining CARS-extracted bands with 1D-CNN achieved the best performance: in the calibration set, $R_{C}^{2}$ and RMSEC were 0.8347 and 0.2768, respectively; in the prediction set, $R_{P}^{2}$ and RMSEP were 0.8205 and 0.2607, with an RPD_P_ of 2.3853, the highest of all models. For further comparison, a 1D-CNN model using the full spectrum was developed as a baseline. The full-spectrum model showed calibration set $R_{C}^{2}$ and RMSEC of 0.8817 and 0.2241, and prediction set $R_{P}^{2}$, RMSEP, and RPD_P_ of 0.8238, 0.2657, and 2.4079, respectively, slightly outperforming the CARS-1D-CNN model. Nevertheless, the CARS algorithm achieved a substantial reduction in input variables—from 256 to only 19 bands—at a minimal cost to accuracy. This significantly enhances model simplicity and computational efficiency, which is of considerable practical value for developing portable real-time detection instruments.

**Figure 8:**
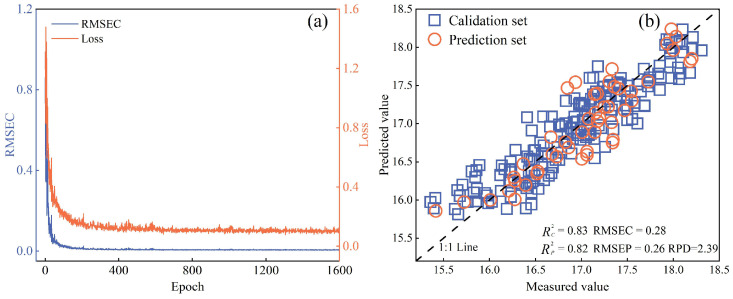
Training process and measured-predicted scatter plot of the CARS–1D-CNN model. (**a**) Changes in RMSEC and loss during training; (**b**) Scatter plot of predicted vs. measured values of the model.

Figure 8 shows the training process of the CARS–1D-CNN model, showing the trends of loss and RMSEC over epochs, as well as the scatter plot of measured versus predicted values under optimal parameter settings. As shown in Figure 8a, both loss and RMSEC decreased continuously with increasing epochs, reaching lower levels by the 112th epoch, indicating good model fitting; subsequent training primarily served to fine-tune parameters for performance optimization. In Figure 8b, the scatter plot demonstrates that the predicted values closely align with the measured values along the diagonal, indicating a strong linear correlation and further validating the reliability of the constructed KCQI predictive model.

### 3.7. Visualization of KCQI

**Figure 9:**
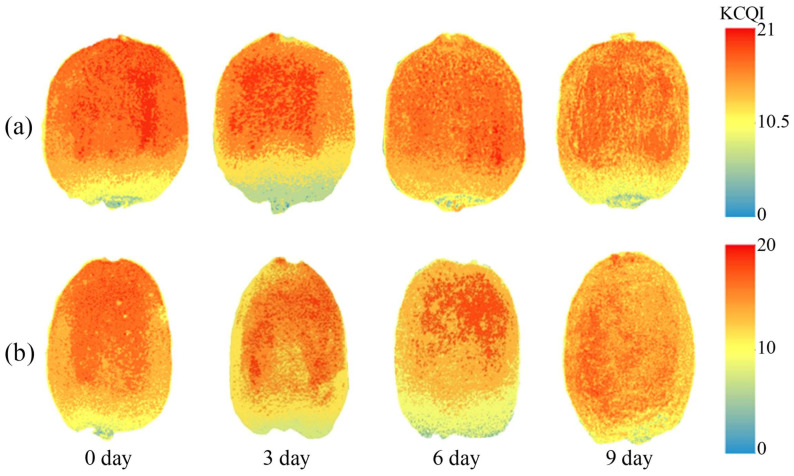
Visualization of KCQI. (**a**) ‘Xuxiang’ and (**b**) ‘Cuixiang’.

To visualize KCQI dynamics during shelf life, the developed CARS–1D-CNN model was implemented for pixel-wise KCQI prediction and spatial visualization (Figure 9). As shelf life progressed, both cultivars exhibited gradual chromatic shifts from dark red to red-yellow in pseudocolor maps, with high-KCQI domains (predominantly red) occupying most fruit-surface pixels. The adjacent colorbar specifies KCQI value ranges for corresponding pixels. These findings demonstrate that HSI-based KCQI spatial maps not only facilitate real-time monitoring of kiwifruit quality progression but also provide critical data foundations for the development of precision quality-sensing instruments.

## 4. Conclusions

To our knowledge, this study is the first to employ the HSI and deep learning to assess the comprehensive quality of kiwifruit during shelf life. Our findings, confirmed by Pearson correlation analysis, revealed significant correlations among quality parameters (SSC, firmness, L*, a*, b*, and chroma) of kiwifruit at different shelf life. Meanwhile, the most representative quality parameters (SSC, firmness, L*, b*, chroma) were selected by factor analysis, and the KCQI incorporating multiple parameters was constructed. Furthermore, spectral characteristic bands related to the KCQI were optimized using SPA, CARS, and RFrog, and predictive models for the KCQI were constructed by combining PLSR, RF, and 1D-CNN. The CARS–1D-CNN model achieved the best performance: the calibration set ($R_{C}^{2}$ = 0.8347, RMSEC = 0.2768) and prediction set ($R_{P}^{2}$ = 0.8205, RMSEP = 0.2607), with an RPD_P_ of 2.3853. Finally, the CARS–1D-CNN model was applied for pixel-level prediction on spectral images of kiwifruit, enabling visualization of KCQI distribution throughout shelf life. This study provides a novel method for assessing the quality of kiwifruit at different shelf life and offers technical support for developing rapid quality-testing instruments.

Notably, this study was conducted under controlled conditions using two specific kiwifruit cultivars. While the proposed method demonstrated excellent performance within this framework, its generalizability to fruits from different harvest seasons and orchards or subjected to varying pre-storage conditions requires further investigation. Future work should focus on validating and calibrating the model with larger and more diverse datasets encompassing a wider range of agricultural practices and environmental variations. This step is crucial for enhancing the model’s robustness and advancing its practical application in the commercial grading and quality monitoring of kiwifruit.

## Figures and Tables

**Table 1:** Component score coefficient matrix.

Index	Factor		
	1	2	3
SSC	−0.807	0.302	−0.437
Firmness	0.833	−0.408	0.155
L*	0.869	0.277	0.027
a*	−0.698	0.494	0.504
b*	0.434	0.822	−0.048
Chroma	0.922	0.331	−0.120

**Table 2:** The Characteristic bands selected for KCQI.

Method	Number	Characteristic Bands (nm)
SPA	8	411, 413, 430, 577, 854, 894, 941, 978
CARS	19	423, 430, 450, 518, 587, 592, 647, 657, 677, 680, 723, 725, 746, 784, 815, 899, 930, 933, 952
RFrog	7	411, 413, 562, 713, 715, 718, 815

**Table 3:** Performance of KCQI predictive models constructed using characteristic bands.

Model	Calibration Set		Prediction Set		${RPD}_{P}$
	$R_{C}^{2}$	RMSEC	$R_{P}^{2}$	RMSEP	
CARS-PLSR	0.8226	0.2868	0.8115	0.2654	2.3279
CARS-RF	0.8201	0.2889	0.8025	0.2739	2.2738
**CARS–1D-CNN**	**0.8347**	**0.2768**	**0.8205**	**0.2607**	**2.3853**
SPA-PLSR	0.7704	0.3113	0.7692	0.2922	2.1038
SPA-RF	0.8447	0.2683	0.8061	0.2714	2.2948
SPA–1D-CNN	0.8187	0.2899	0.81701	0.2622	2.3624
RFrog-PLSR	0.7818	0.3035	0.7799	0.2854	2.1538
RFrog-RF	0.8272	0.2830	0.7918	0.2793	2.2147
RFrog–1D-CNN	0.8146	0.2932	0.8015	0.2731	2.2680

## Data Availability

The original contributions presented in this study are included in the article. Further inquiries can be directed to the corresponding authors.
